# Intratumoral microbiota: implications for colorectal cancer pathogenesis and therapy

**DOI:** 10.3389/fimmu.2026.1832875

**Published:** 2026-09-15

**Authors:** Junhong Liu, Zhonghua Qi

**Affiliations:** 1 Affiliated Hospital of Gansu University of Traditional Chinese Medicine, Lanzhou, China; 2 Gansu University of Traditional Chinese Medicine, Lanzhou, China

**Keywords:** colorectal cancer, diagnostic biomarker, immune response, intratumoral microbiota, metabolic reprogramming, tumor microenvironment

## Abstract

CRC is the third most prevalent malignancy worldwide and the second leading cause of cancer-related mortality. According to GLOBOCAN 2022 estimates, approximately 1.93 million new CRC cases and 900,000 CRC-related deaths occur annually worldwide. Incidence rates continue to rise in developing countries, driven by the westernization of dietary patterns, sedentary lifestyles, and increasing obesity prevalence, with a notable trend toward younger-onset disease. In recent years, mounting evidence has implicated the microbiota as a critical contributor to CRC initiation and progression. Distinct from the fecal microbiome, the intratumoral microbiome constitutes an independent, low-biomass microbial community predominantly composed of bacteria, with additional fungal, viral, and archaeal components. Microbial communities within the TME promote CRC development and progression by modulating host immune responses, metabolic pathways, and tumor cell proliferation and metastasis. *Fusobacterium nucleatum*, ETBF, polyketide synthase genotoxicity island-harboring *Escherichia coli* and *Parvimonas micra* represent the most well-substantiated candidate oncomicrobes, whose carcinogenic activities involve multiple intertwined pathways, including chronic inflammation, genotoxic DNA damage, immune evasion, metabolic reprogramming, and non-coding RNA regulation. Furthermore, intratumoral microbiota may serve as early diagnostic biomarkers for CRC, offer novel therapeutic targets, and provide potential biological rationale for prognostic prediction. Microbiome-based diagnostic biomarkers, such as multi-bacterial fecal/tissue panels, have demonstrated sensitivity comparable to or superior to the fecal immunochemical test in select retrospective cohorts; however, large-scale, multicenter, prospective validation remains necessary. Strategies targeting intratumoral bacteria — including antibiotics, bacteriophages, phage-guided nanocarriers, and FMT — remain predominantly in the preclinical stage. Although phase I/II trials of FMT for immunotherapy sensitization have yielded encouraging results, small sample sizes and substantial heterogeneity preclude its near-term adoption as a standard-of-care recommendation. This review systematically summarizes the latest advances in understanding the relationship between intratumoral microbiota and CRC, critically appraises the strength of evidence across different hierarchical levels, evaluates the consistency and controversies surrounding major mechanistic hypotheses, addresses methodological challenges inherent to low-biomass microbiome research, and discusses the translational potential in CRC diagnosis, therapy, and prognostic prediction.

## 1 Introduction

Colorectal cancer (CRC) ranks as the third most commonly diagnosed malignancy and the second leading cause of cancer-related death globally. According to the latest GLOBOCAN 2022 statistics, approximately 1.93 million new CRC cases and 900,000 deaths were recorded worldwide in 2022, ranking third and second among all malignancies in incidence and mortality, respectively (1). In developed countries, CRC mortality has been declining owing to the widespread adoption of early screening and standardized treatment; however, in many developing nations, both incidence and mortality rates continue to escalate in parallel with lifestyle westernization (2). Notably, CRC has exhibited a marked trend toward younger-onset disease — incidence among individuals under 50 years of age has been increasing at an annual rate of approximately 2% across multiple high-income countries, a phenomenon potentially attributable to gut microbiota alterations shaped by early-life exposures, including cesarean delivery, antibiotic use, and infant dietary patterns (3). The pathogenesis of CRC arises from the interplay between genetic and environmental factors, with environmental determinants such as unhealthy dietary habits, physical inactivity, and tobacco use playing pivotal roles in disease initiation (4). Recent phenome-wide Mendelian randomization analyses have further substantiated body mass index as a causal mediator linking modifiable lifestyle factors to CRC risk, offering a quantitative framework for prioritizing intervention targets (5). Emerging evidence has revealed a pivotal role for the microbiota in CRC pathogenesis. Within the hypoxic and immunosuppressive tumor microenvironment (TME) characteristic of CRC, microorganisms participate in disease progression by modulating immune responses and altering metabolic pathways (6–8).

Intratumoral microbiota refers to the microbial communities residing within the TME, encompassing bacteria, viruses, fungi, and other microorganisms (9). Unlike the fecal or luminal microbiome, the intratumoral microbiome is characterized by low biomass, with bacterial DNA copy numbers per gram of tissue typically 2–4 orders of magnitude lower than those in fecal samples (10). These microorganisms can colonize tumor tissues and influence tumorigenesis, progression, and therapeutic responses through multiple mechanisms, including metabolite production, immune modulation, and direct interactions with host cells (11). The origins of intratumoral microbiota can be attributed to three principal routes (12): (1) Mucosal barrier breach: In gastrointestinal malignancies such as CRC and pancreatic cancer, as well as cancers of organs exposed to external cavities (e.g., lung and cervical cancers), mucosal surface-colonizing microorganisms can invade tumor tissues through compromised mucosal barriers. (2) Adjacent tissue migration: Microorganisms have been detected in organs traditionally considered sterile, such as the pancreas, with their composition closely resembling that of neighboring tumor tissues. The immunosuppressive and hypoxic TME may further facilitate microbial colonization. (3) Hematogenous dissemination: Microorganisms from the oral cavity, intestine, and other sites can disseminate to distant tumor locations via the bloodstream. Studies have identified *Bacteroides* species in canine mammary tumors as well as in the oral cavity and intestine (13), suggesting that microorganisms can achieve distant colonization through circulatory spread.

Advances in next-generation sequencing technologies have driven pivotal breakthroughs in intratumoral microbiome research. Nejman et al. systematically analyzed 1,526 tumors and their adjacent normal tissues, providing the first comprehensive evidence that seven distinct cancer types harbor characteristic intratumoral microbial profiles, which influence cancer progression through interactions with tumor and immune cells (14). However, reanalysis of a subset of Nejman et al.’s data by Salzberg’s group in 2023 raised contamination concerns, suggesting that several putative intratumoral microbial signals may have originated from host mitochondrial/chloroplast sequences or reagent background — a controversy that remains unresolved. This underscores the need for cautious interpretation of intratumoral microbiome findings (15). Subsequent studies have demonstrated that high abundances of fungi such as *Candida* and *Saccharomyces cerevisiae* in gastrointestinal tumors are closely associated with carcinogenesis (16). In-depth investigations have revealed intricate host–microbe interaction networks at the molecular, cellular, and spatial levels, underscoring the significance of microorganisms in CRC progression (17). This review systematically summarizes the current knowledge regarding enriched intratumoral microbiota in CRC and their pathological implications, critically evaluates the strength of evidence at different hierarchical levels, assesses the consistency and controversies of mechanistic hypotheses, and addresses methodological limitations of low-biomass microbiome studies, with the aim of providing a theoretical framework for developing CRC-associated tissue microbiome-based biomarkers for diagnosis, therapy, and prognostic prediction.

## 2 Intratumoral microbiota in colorectal cancer

The application of metagenomic sequencing has unveiled the critical role of the tumor microbiome within the CRC TME (18). *Fusobacterium* abundance was found to be significantly elevated in liver-metastatic CRC but not in primary hepatocellular carcinoma (19). Moreover, intratumoral *E. coli* has been shown to be enriched in tumor tissue, highlighting the potential contribution of the tumor microbiome to CRC progression. However, these pro-tumorigenic descriptions are primarily based on colonization–tumorigenesis associations in murine models and *in vitro* mechanistic experiments; causal evidence at the human level remains relatively limited. The intratumoral microbiome exhibits considerable heterogeneity, with its composition undergoing dynamic shifts during the adenoma-to-carcinoma transition in CRC (19). Studies have indicated that *Fusobacterium*, *Bacteroides*, *Parvimonas*, and *Prevotella* are enriched in tumor tissues (19). In a study of 294 paired tumor and adjacent tissue biopsies, the abundances of *Fusobacterium*, *Streptococcus*, and *Proteobacteria* were increased in CRC tumor tissues, whereas *Firmicutes* abundance was decreased (20). Furthermore, intratumoral microbiome composition is associated with tumor anatomical location, with significant differences observed between ascending and descending colon cancer samples (21), yet within individual tumors, the microbiome displays a relatively homogeneous distribution (22).

### 2.1 Intratumoral microbial species in CRC

Microbial community diversity is typically assessed using α-diversity (species richness within a single sample) and β-diversity (compositional dissimilarity across samples). Compared with normal tissues, the intratumoral microbiome in CRC is characterized by decreased α-diversity and increased β-diversity (23). In CRC tissues, the relative abundances of Proteobacteria, Fusobacteria, Campylobacterota, and Spirochaetota are increased, whereas those of Bacteroidota, Firmicutes, Verrucomicrobiota, Actinobacteriota, and Euryarchaeota are decreased (24). **Table 1** summarizes the key intratumoral bacterial species in CRC and their oncogenic mechanisms. *Fusobacterium nucleatum* is among the most frequently detected bacteria in CRC patients, with its DNA predominantly localized within tumor cells (25). Its abundance is markedly higher in stage III/IV patients than in stage I patients, suggesting a close association between bacterial abundance and CRC prognosis (19). Multiple studies employing quantitative polymerase chain reaction, 16S ribosomal RNA (rRNA) gene sequencing, and metagenomic analyses have confirmed the high abundance of *Fusobacterium* in CRC patients (19, 26, 27). Furthermore, *F. nucleatum* DNA concentration is inversely correlated with patient survival an association potentially attributable to its capacity to promote perineural invasion and vascular tumor embolization (28). Microsatellites are short tandem repeat DNA sequences, typically comprising 2–6 base pairs. Microsatellite instability-high (MSI-H) status is closely linked to CRC pathogenesis and generally portends a favorable prognosis with enhanced immunotherapy responsiveness, whereas microsatellite stable (MSS) tumors are associated with poorer prognosis and diminished immunotherapeutic efficacy (29). Beyond classical mismatch repair gene defects, EPCAM pathogenic variants in familial Lynch syndrome–associated CRC have recently been linked to distinct tumor microenvironmental features, underscoring the genotype-dependent shaping of the immune microenvironment (30). *Fusobacterium* modulates immune responses within the TME (31); in MSI-H CRC, it may promote tumor progression by suppressing adaptive antitumor immunity, whereas in MSS CRC, it predominantly exerts a pro-inflammatory effect (31). Studies by Hamada et al. and Oh et al. have suggested that the prognostic impact of *F. nucleatum* is contingent upon tumor location and MSI status, cautioning against its designation as a universal prognostic factor (32). Additionally, the fungal community composition within tumor tissues undergoes notable alterations, with elevated *Malassezia* abundance and significantly reduced *Saccharomyces* and *Pneumocystis* levels (33). However, given that the absolute abundance of fungi within tumors is substantially lower than that of bacteria, their signals are highly susceptible to contamination artifacts and database biases; consequently, these mycobiome findings require independent cohort validation.

**Table 1:** Key intratumoral bacterial species in CRC and their oncogenic mechanisms.

Microorganism	Abundance in CRC	Effect on TME	Clinical relevance
*Fusobacterium nucleatum*	Markedly elevated	Suppresses CD8+ T cells; recruits MDSCs	Poor prognosis; inversely correlated with survival
*Enterotoxigenic* Bacteroides fragilis (ETBF)	Elevated in polyps and CRC	Pro-inflammatory; DNA damage via ROS	Higher detection in advanced-stage CRC
*pks+ Escherichia coli*	Enriched in tumor tissue	Genomic instability, APC mutations	Drives early somatic mutations in CRC
*Peptostreptococcus anaerobius*	Elevated in CRC	Promotes cell proliferation	Associated with tumor growth
*Parvimonas micra*	Elevated in CRC	Promotes proliferation and invasion	Potential biomarker
*Bifidobacterium* spp.	Reduced in CRC	Enhances anti-tumor immunity	Improves anti-CD47 immunotherapy efficacy
*Lachnospiraceae*	Reduced in CRC	Relieves CD8+ T cell suppression	Protective role

### 2.2 Intratumoral microbial dynamics during carcinogenesis

The dynamic evolution of intratumoral microbial communities is intimately linked to CRC progression. At the adenoma stage, highly variable microorganisms are predominantly represented by Proteobacteria (43.14%) (34). During adenoma-to-carcinoma progression, the proportion of Proteobacteria progressively declines and is supplanted by Firmicutes (54.00%), accompanied by a concomitant reduction in the number of highly variable intratumoral microbes (35). *F. nucleatum* is markedly enriched in CRC tissues, with its abundance increasing from 5.9% in the adenoma stage to 81.8% in advanced CRC (36). Among the fungal community, the proportions of *Candida* and *Saccharomyces cerevisiae* are relatively low in early-stage CRC but increase substantially in stage IV tumors (16). Invasion of intestinal microorganisms through compromised gut mucosal barriers into colorectal tissues constitutes a major source of intratumoral microbiota in CRC (7). Further transcriptomic analyses of 807 CRC samples revealed that 17 intratumoral bacterial species originated from the oral microbiome (37). Among these, orally derived *F. nucleatum* colonizes tumor tissues by recognizing Gal-GalNAc glycosylation structures overexpressed in CRC tissues (38). Furthermore, intratumoral *E. coli* can enter the bloodstream through the compromised gut vascular barrier and colonize the liver, thereby promoting the establishment of a “pre-metastatic niche” that facilitates hepatic metastasis of CRC (7).

## 3 Mechanisms of intratumoral microbiota-driven CRC

CRC pathogenesis is intimately associated with multiple intratumoral microorganisms, among which *F. nucleatum*, *Bacteroides fragilis* (ETBF), and pks⁺ *E. coli* constitute the principal pathogenic consortium (39). These microorganisms influence CRC initiation and progression through diverse mechanisms, including regulation of tumor cell apoptosis, promotion of metastasis, remodeling of the immune microenvironment, and induction of DNA damage (40). The impact of intratumoral microbiota on CRC pathogenesis can be conceptualized along four intertwined core axes: chronic inflammation, immune suppression, metabolic reprogramming, and epigenetic/ncRNA regulation. Along the inflammatory axis, pathogenic bacteria activate nuclear factor-kappa B (NF-κB) via the Toll-like receptor (TLR)–myeloid differentiation primary response 88 (MyD88)–interleukin-1 receptor-associated kinase 1/4 (IRAK1/4)–tumor necrosis factor receptor-associated factor 6 (TRAF6) cascade, inducing the release of pro-inflammatory cytokines including interleukin-6 (IL-6), tumor necrosis factor-α (TNF-α), and IL-1β Along the immunosuppressive axis, expansion of myeloid-derived suppressor cells (MDSCs), M2-polarized macrophages, and regulatory T cells (Tregs), coupled with functional suppression of CD8⁺ T cells and natural killer (NK) cells, establishes an immune evasion network mediated by IL-10, transforming growth factor-β (TGF-β), and C-C motif chemokine ligand 28 (CCL28) (41, 42). Along the metabolic axis, tumor cells rely on enhanced glycolysis and amino acid metabolism — particularly glutamine — while microbial metabolites such as succinate, bile acids, and short-chain fatty acids (SCFAs) further remodel the cellular energy and redox landscape (43). Along the epigenetic and ncRNA axis, microorganisms alter host microRNA (miRNA)/long non-coding RNA (lncRNA) networks, thereby regulating post-transcriptional expression of key genes (44). These axes do not operate in isolation but are mutually coupled through hub signaling nodes such as NF-κB, β-catenin, and mechanistic target of rapamycin (mTOR), collectively driving CRC initiation and progression (45). **Figure 1** illustrates the mechanisms by which intratumoral microbiota influence CRC development and progression.

**Figure 1:**
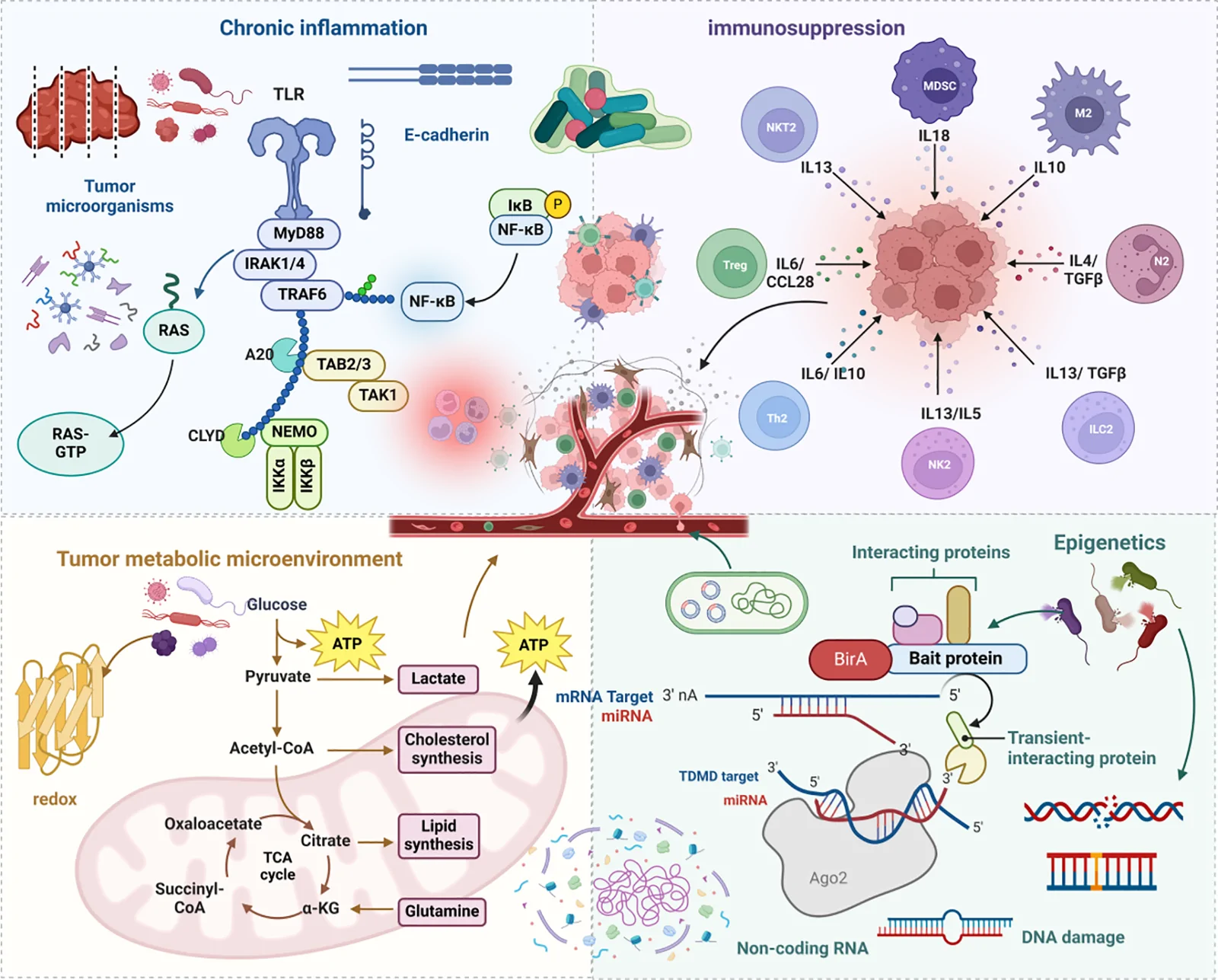
Schematic overview of the mechanisms by which intratumoral microbiota drive colorectal cancer initiation and progression. This figure integrates the roles of intratumoral microbiota across four intertwined pathogenic axes — chronic inflammation, immune suppression, metabolic reprogramming, and epigenetic regulation — in CRC development. Chronic inflammation axis: Intratumoral microbiota induce chronic inflammatory responses in host cells by activating the TLR–MyD88–IRAK1/4–TRAF6 signaling cascade, which promotes NF-κB activation and subsequent release of pro-inflammatory cytokines (e.g., IL-6, TNF-α, IL-1β), establishing a chronic inflammatory microenvironment conducive to CRC initiation and progression. Immune suppression axis: Intratumoral microbiota modulate immunoregulatory cell populations, including myeloid-derived suppressor cells, M2-polarized macrophages, type 2 natural killer T cells, and group 2 innate lymphoid cells, promoting the secretion of immunosuppressive factors such as IL-10, IL-13, and TGF-β, thereby attenuating T cell-mediated antitumor responses. The regulatory T cell- and T helper 2 cell-mediated IL-6/CCL28 axis further facilitates immune evasion, enabling CRC cell survival and dissemination. Metabolic reprogramming axis: Intratumoral microbiota sustain the high proliferative capacity of tumor cells through metabolic reprogramming. Glucose is converted to pyruvate via glycolysis and subsequently to lactate to maintain ATP production, while the tricarboxylic acid cycle generates metabolic intermediates such as citrate and α-ketoglutarate that fuel biosynthetic demands of CRC cells. Epigenetic and post-transcriptional regulation axis: Intratumoral microbiota modulate miRNA–mRNA targeting interactions, thereby altering gene expression and influencing post-transcriptional regulatory networks in CRC cells.

### 3.1 Intratumoral microbiota-induced inflammatory responses

Intratumoral microbiota play a pivotal role in the inflammation-to-cancer transition of CRC by reshaping the intestinal immune milieu, inducing chronic inflammatory responses, and modulating cytokine expression and signaling pathway activation, thereby promoting CRC initiation and metastasis (40). Non-toxigenic *B. fragilis* (NTBF) and ETBF, as dominant intratumoral colonizers, exert significant pro-inflammatory effects in CRC pathogenesis (46). NTBF is significantly enriched in the microenvironment of precancerous colonic polyps, with upregulated expression of lipopolysaccharide (LPS) biosynthesis genes that activates TLR4-mediated inflammatory signaling (47). This conclusion is primarily supported by metagenomic profiling of human early-stage adenoma samples complemented by murine colonization experiments, representing a “human association plus animal model validation” level of evidence. This inflammatory milieu not only promotes colonic polyp growth but also provides favorable conditions for the colonization of oncogenic microorganisms such as pks⁺ *E. coli* and *F. nucleatum*, thereby accelerating CRC development (47). F. *nucleatum* activates carcinoembryonic antigen-related cell adhesion molecule 1 (CEACAM-1)-dependent protein tyrosine phosphorylation signaling in CRC stem cells, inducing the upregulation of pro-inflammatory mediators including C-X-C motif chemokine ligand 1 (CXCL1), CXCL8, and NF-κB, thereby amplifying pro-inflammatory and oncogenic effects (48). ETBF-secreted *B. fragilis* toxin (BFT) disrupts the E-cadherin/β-catenin complex, leading to E-cadherin proteolysis, nuclear translocation of β-catenin, and subsequent promotion of cell proliferation (49). Concurrently, BFT suppresses inhibitor of NF-κB activity, activating the canonical NF-κB pro-inflammatory signaling pathway and triggering a cytokine release cascade centered on NF-κB signaling, which further propels CRC initiation and progression (50, 51). *F. nucleatum* also activates the TLR4/MYD88/NF-κB signaling axis, suppresses RAS p21 protein activator 1 (RASA1) activity, and consequently activates the KRAS signaling pathway, promoting CRC cell proliferation and survival (41).

Conversely, certain intratumoral or intestinal microorganisms counteract tumorigenesis through anti-inflammatory mechanisms, with the underlying pathways varying across different bacterial species. NTBF, through its surface polysaccharide A, activates the TLR2/Treg axis, inducing IL-10 production by CD4⁺ T cells, thereby alleviating colonic inflammation and suppressing colitis-associated CRC development in certain models (52). Within the colonic microecosystem, butyrate — the principal molecule that inhibits the NOD-like receptor protein 3 (NLRP3) inflammasome and reduces IL-18 and IL-1β release — is primarily produced by butyrate-generating bacteria (e.g., *Faecalibacterium prausnitzii*, *Roseburia* spp., *Butyricicoccus*) via the butyryl-CoA:acetate CoA-transferase pathway. As a histone deacetylase (HDAC) inhibitor and G-protein-coupled receptor 43/109A (GPR43/GPR109A) agonist, butyrate suppresses macrophage activation and induces Treg expansion, thereby exerting anti-inflammatory and antitumor effects (53, 54). The evidence for the NTBF–PSA–Treg/IL-10 axis is predominantly derived from murine models, and the immunological consequences of NTBF enrichment in human CRC warrant further investigation (55). Moreover, the pro-inflammatory and anti-inflammatory roles of microbiota in CRC progression exist in dynamic equilibrium. When this balance is disrupted, pro-inflammatory microorganisms predominate, and chronic inflammation may further induce gene mutations, tumor suppressor inactivation, and other oncogenic cascades, ultimately facilitating tumor cell invasion, metastasis, and the development of therapeutic resistance.

### 3.2 Intratumoral microbiota and the tumor immune microenvironment

The tumor immune microenvironment (TIME) encompasses the tumor cells and their surrounding immune cells, immune factors, immunomodulatory molecules, and other immune response-associated cellular and molecular components that collectively constitute a specialized niche. Intratumoral microbiota play an instrumental role in CRC initiation and progression by modulating the TIME (56, 57). Microbial community diversity is closely correlated with immune cell composition within the TIME, and this relationship exhibits bidirectional regulatory characteristics. Microbiota can influence CRC progression and prognosis by either enhancing pro-tumorigenic immune responses or suppressing antitumor immunity (56, 57). **Figure 2** illustrates the mechanisms by which intratumoral microbiota modulate the TIME. Understanding how intratumoral microbiota reshape the TIME through immune regulation is essential for elucidating their oncogenic mechanisms.

**Figure 2:**
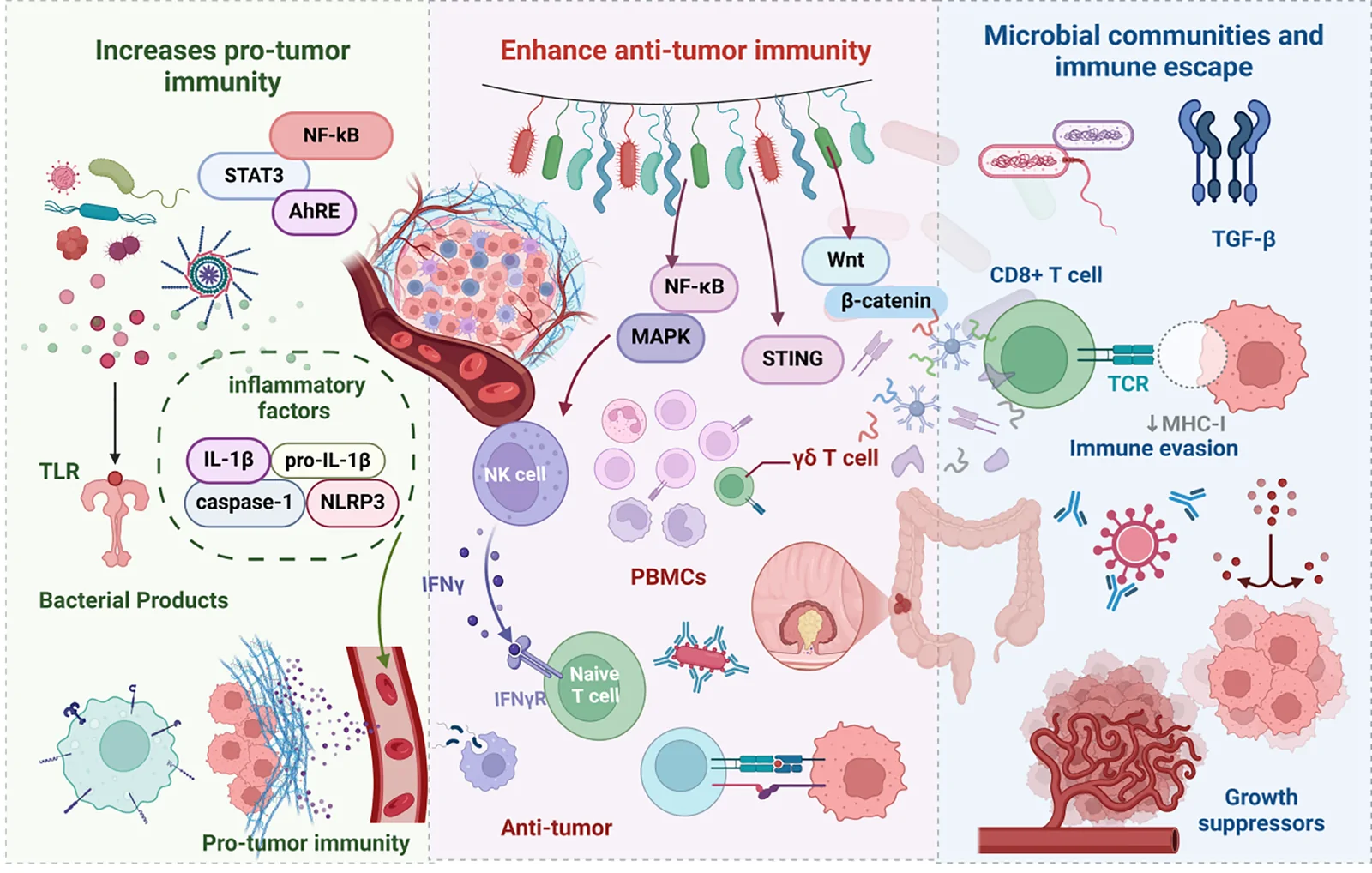
Schematic illustration of intratumoral microbiota-mediated modulation of the tumor immune microenvironment in CRC. This figure delineates the dual immunomodulatory roles of intratumoral microbiota in CRC. Pro-inflammatory and pro-tumorigenic signaling: Intratumoral microbiota activate Toll-like receptors, initiating downstream NF-κB, STAT3, and aryl hydrocarbon receptor signaling pathways. This triggers inflammatory responses characterized by the release of IL-1β, pro-IL-1β, caspase-1, and NOD-like receptor protein 3 inflammasome components, thereby reinforcing an inflammatory microenvironment that favors tumor growth and immune evasion. Antitumor immune activation: Certain intratumoral microbiota activate host antitumor immunity by engaging NF-κB, mitogen-activated protein kinase, stimulator of interferon genes, and Wnt/β-catenin signaling pathways, which enhance the cytotoxic activity of natural killer cells, γδT cells, and peripheral blood mononuclear cells. Interferon-γ secretion promotes naïve T cell priming, thereby augmenting systemic antitumor immunity. Immune evasion: Intratumoral microbiota facilitate tumor cell escape from immune surveillance by activating the transforming growth factor-β signaling pathway, which suppresses CD8⁺T cell cytotoxicity and diminishes T cell recognition of tumor cells, ultimately enabling immune evasion and promoting tumor growth. This figure underscores the bidirectional nature of intratumoral microbiota in CRC immune regulation: they can simultaneously activate immune defenses to restrain tumor growth and assist tumor cells in evading immune surveillance to promote cancer progression.

#### 3.2.1 Enhancement of pro-tumor immunity

Regarding pro-tumor immunity, intratumoral microbiota promote CRC development by inducing the recruitment and activation of immune cells, thereby amplifying pro-inflammatory responses within the TME. *Porphyromonas gingivalis* recruits bone marrow-derived immune cells and further upregulates the expression of inflammatory mediators including NLRP3, caspase-1, IL-1β, and pro-IL-1β, significantly exacerbating CRC progression (58). *Candida albicans*, as a fungal pathogen, induces macrophage glycolysis and IL-7 secretion, activating the aryl hydrocarbon receptor (AhR)/signal transducer and activator of transcription 3 (STAT3) signaling pathway to promote IL-22 secretion by innate lymphoid cells (ILCs), thereby driving intestinal epithelial hyperplasia and pro-tumorigenic immune responses (59). *F. nucleatum* modulates cytochrome P450 2J2 (CYP2J2) expression via TLR4 signaling, contributing to carcinogenesis. Its metabolite, 12,13-epoxyoctadecenoic acid (12,13-EpOME), enhances tumor cell invasiveness by promoting epithelial–mesenchymal transition (EMT), further aggravating tumor metastasis (41, 60). Additionally, *F. nucleatum* recruits MDSCs to the TME through activation of NF-κB-mediated inflammatory responses. These MDSCs suppress T cell proliferation and induce T cell apoptosis, diminishing immune surveillance efficacy (61).

#### 3.2.2 Enhancement of antitumor immunity

With respect to antitumor immunity, intratumoral microbiota suppress tumorigenesis and progression by promoting the activation of specific immune cell subsets and enhancing immune surveillance. Lachnospiraceae family bacteria degrade lysophospholipids, attenuating their suppressive effects on CD8⁺ T cells and thereby preserving immune surveillance capacity (62). However, the protective effects of *H. hepaticus* have been observed only in specific mouse strains and short-term models, and the direction of its role in human CRC remains unclear — the same genus may produce opposite effects in different host backgrounds and disease stages, a context-dependent phenomenon commonly encountered in intratumoral microbiology research. These microorganisms not only enhance local immune responses and augment tumor antigen presentation but also promote the functional recovery of immune cells within the TME.

*Bifidobacterium* species activate the stimulator of interferon genes signaling pathway, promoting interferon-β secretion by dendritic cells (DCs) and enhancing adaptive immune responses, thereby augmenting antitumor immunity. Studies have demonstrated that *Bifidobacterium* enhances the efficacy of anti-CD47 antibody therapy, showing particular promise in immune checkpoint inhibitor (ICI)-based treatments (63). However, this evidence is derived from murine tumor models and has not yet been validated in randomized controlled trials involving human CRC patients, further indicating that microorganisms can effectively suppress tumor growth by enhancing immune system recognition and clearance of tumors. *Bifidobacterium adolescentis* plays a unique role in the TIME. *B. adolescentis* suppresses the activation of cancer-associated fibroblasts (CAFs) by modulating the Wnt/β-catenin signaling pathway, reducing their supportive role in tumor cell maintenance (64). *B. adolescentis* promotes growth arrest-specific protein 1 expression in CD143⁺ CAFs, thereby suppressing CRC development (64).

#### 3.2.3 Microbial communities and immune evasion

Disruption of intratumoral microbiome homeostasis is considered a key factor in tumor immune evasion. Notably, such dysbiosis is frequently a consequence of tumor formation rather than a sole causative factor — a distinction that warrants careful consideration in causal inference. As TME complexity increases, specific microorganisms induce immune tolerance and suppress immune cell function through multiple mechanisms, thereby promoting tumor immune evasion (65). *F. nucleatum* promotes TGF-β secretion within the TME, suppressing CD8⁺ T cell effector functions, attenuating immune surveillance, and inducing immune tolerance. This mechanism enables tumor cells to escape host immune surveillance and promotes CRC progression (66). Furthermore, *F. nucleatum* upregulates programmed death-ligand 1 (PD-L1) expression on tumor cell surfaces, further potentiating tumor cell-mediated T cell suppression. This effect not only impairs T cell antitumor function but also exacerbates the degree of immune evasion, allowing tumor cells to survive and expand under immune system attack (65). The occurrence of this immune evasion process reveals the profound influence of the microbiome on tumor immune escape mechanisms. Furthermore, the Fap2 adhesin of F. nucleatum directly engages the TIGIT inhibitory receptor on NK cells and T cells, shielding tumor cells from cytotoxic immune attack — a mechanism complementary to PD-L1 upregulation that establishes a multilayered immune evasion network (38).

Enhanced Treg functionality and accumulation represent another potential mechanism of tumor immune evasion. Tregs suppress effector T cell activity and antitumor immune responses, providing tumor cells with opportunities for immune escape (67). Conversely, alterations in microbiome composition can also enhance antitumor immunity: gut commensal bacteria can imprint T cell plasticity — inducing TH17-to-TH1 cell conversion — that potentiates immune checkpoint blockade and enables immune-mediated tumor control (68). In summary, intratumoral microbiota not only enhance pro-tumorigenic immune responses to promote tumor initiation and progression but also profoundly influence the dynamic equilibrium of the TIME through modulation of immune evasion mechanisms. Microbial regulation of immune cell function, cytokine secretion, and immune evasion induction provides multiple avenues for tumor immune escape.

### 3.3 Intratumoral microbiota and tumor energy metabolism reprogramming

Intratumoral microbiota regulate tumor cell energy metabolism across multiple intertwined axes, encompassing glycolysis, amino acid metabolism (particularly glutamine metabolism), lipid metabolism, redox homeostasis, and microbially derived SCFA/bile acid metabolism, collectively influencing CRC metabolic reprogramming and consequently tumor growth, proliferation, and metastasis (69). The interplay between microbial and host metabolism drives CRC initiation and progression not only by altering tumor cell metabolic patterns but also by modulating immune system and microenvironment metabolic equilibria.

#### 3.3.1 Glycolysis

Glycolysis is widely recognized as a primary metabolic pathway in tumor cells, not only fulfilling the energy and macromolecular demands of rapidly proliferating cells but also enhancing tumor cell tolerance to environmental stressors (such as radiotherapy and chemotherapy), thereby facilitating tumor growth and metastasis (69). Excessive reliance on glycolysis triggers a cascade of metabolic reprogramming effects within the TME, altering tumor cell energy metabolism and intensifying competition for nutrient resources between tumor cells and tumor-infiltrating cells. Recent studies have revealed that intratumoral microbiota participate in this metabolic reprogramming process by influencing tumor cell metabolic pathways, further driving CRC growth, metastasis, and therapeutic resistance (70). *F. nucleatum* enhances glycolytic activity in CRC cells by inducing angiopoietin-like protein 4 expression; Elevated ANGPTL4 subsequently upregulates glucose transporter 1 expression, increasing glucose uptake and establishing a positive feedback loop that further promotes *F. nucleatum* colonization, thereby accelerating tumor cell proliferation and metastasis (71).

#### 3.3.2 Lipid metabolism and redox balance

The proliferative demands of tumor cells engender dependence on lipid metabolism, with fatty acid synthesis and fatty acid oxidation being highly upregulated. These lipids not only provide energy but also serve as building blocks for tumor cell membrane synthesis. Moreover, alterations in lipid metabolism are closely associated with tumor cell tolerance to oxidative stress, enhancing their adaptive capacity to environmental pressures. *Peptostreptococcus anaerobius* alters CRC cell metabolic pathways through its interactions with host immune responses (72). *P. anaerobius* activates TLR2 and TLR4, inducing reactive oxygen species generation and upregulating sterol regulatory element-binding protein 2 expression to promote cholesterol synthesis, ultimately enhancing tumor cell proliferation (72). Redox reactions play a central role in tumor cell metabolism, with critical functions in energy conversion and cell survival. *Lactobacillus reuteri* and its metabolite reuterin are downregulated in both murine and human CRC tissues. Reuterin suppresses colon cancer cell proliferation and survival by modulating the intestinal redox balance (73). This finding indicates that specific intestinal probiotics and their metabolites may play important roles in regulating redox status, thereby suppressing tumor cell proliferation and improving the TME.

#### 3.3.3 Amino acid metabolism

Amino acid metabolism constitutes the second major axis of tumor metabolic reprogramming alongside glycolysis, with glutamine often referred to as the “second glucose” of CRC cells. CRC cells avidly uptake glutamine via the high-affinity transporter solute carrier family 1 member 5/alanine-serine-cysteine transporter 2 (SLC1A5/ASCT2). Glutaminase converts glutamine to glutamate, which is subsequently transformed into α-ketoglutarate by glutamate dehydrogenase or transaminases, feeding into the tricarboxylic acid (TCA) cycle to provide carbon skeletons for macromolecular synthesis while supporting redox homeostasis through glutathione production (74–76). This “glutamine addiction” is particularly pronounced in MYC- and KRAS-mutant CRC (69).

Intratumoral microbiota and glutamine metabolism engage in multilayered interactions. First, *F. nucleatum* infection upregulates SLC1A5 expression in CRC cells, increasing glutamine uptake and activating the mechanistic target of rapamycin complex 1/ribosomal protein S6 kinase 1 (mTORC1/S6K1) pathway, thereby promoting cell proliferation; this process simultaneously increases α-KG and succinyl-CoA production, directing carbon flux into the TCA cycle in the reverse direction. Second, intratumoral bacterial succinate accumulation — such as succinate secreted by *F. nucleatum* — suppresses CD8⁺ T cell mitochondrial function and migration via the succinate receptor 1, establishing a dual “metabolic–immune” suppression (77). Third, ETBF induces spermine oxidase expression via BFT, promoting polyamine metabolite production and downstream ROS generation through activation of the ornithine/arginine metabolic pathway (78). Fourth, microbially derived tryptophan metabolites (e.g., indole-3-aldehyde and kynurenine) regulate colonic epithelial stem cell homeostasis and Treg differentiation via the AhR. The net effect of AhR activation is context-dependent, varying with specific ligands and tumor stage — with evidence supporting both pro-inflammatory/oncogenic and gut barrier-protective outcomes — exemplifying a classic “double-edged sword” phenomenon (43).

#### 3.3.4 Microbially derived metabolites: SCFAs and bile acids

Beyond direct participation in tumor cell metabolic reprogramming, microbially derived metabolites themselves are important modulators of the CRC microenvironment. SCFAs — primarily acetate, propionate, and butyrate — are produced by butyrate-generating bacteria (*Faecalibacterium*, *Roseburia*, *Butyricicoccus*, etc.) through dietary fiber fermentation. Butyrate exhibits a dual role: in normal colonocytes, it serves as a primary energy source via β-oxidation; in CRC cells dominated by the Warburg effect, β-oxidation of butyrate is impaired, leading to intracellular accumulation where it functions as an HDAC inhibitor, inducing p21/WAF1 expression and promoting apoptosis. This “butyrate paradox” elegantly explains the colonoprotective mechanism of dietary fiber (79). Based on this principle, SCFA delivery systems such as butyrate prodrugs and butyrate-loaded nanoparticles have been proposed as adjunctive CRC therapeutic strategies (43). Bile acid metabolism is equally central to the intratumoral microbiota–CRC interaction network. Primary bile acids (cholic acid and chenodeoxycholic acid), synthesized in the liver and entering the intestine via the biliary tract, undergo deconjugation by bile salt hydrolase (BSH)-expressing bacteria (predominantly *Bacteroides* and *Clostridium* species), followed by 7α-dehydroxylation to generate secondary bile acids, including deoxycholic acid (DCA) and lithocholic acid (LCA) (43). Elevated DCA levels promote colonic epithelial DNA damage and tumorigenesis through epidermal growth factor receptor (EGFR)/STAT3 activation, farnesoid X receptor (FXR) suppression, and oxidative stress, while LCA exhibits complex bidirectional effects through Takeda G-protein-coupled receptor 5 (TGR5) agonistic activity. *F. nucleatum* and *Clostridium histolyticum* can directly alter local bile acid profiles, creating a metabolic microenvironment favorable for tumor growth (43). Collectively, the intratumoral microbiota–metabolic reprogramming axis is not a singular pathway but rather a multidimensional network interwoven across glycolysis, glutamine metabolism, lipid metabolism, redox balance, and SCFA/bile acid metabolism. Therapeutic targeting of this axis necessitates integrated metabolomic and microbiome-based strategies (80).

### 3.4 Intratumoral microbiota and DNA damage

DNA damage is a key driver of CRC pathogenesis. Intratumoral microbiota promote CRC development through direct genotoxic effects or toxin production that damages host cell DNA. pks⁺ *E. coli* represents a major source of DNA damage and, notably, possesses the strongest causal evidence among intratumoral oncomicrobes — long-term co-culture of human intestinal organoids with pks⁺ *E. coli* has directly revealed the characteristic T>N mutational signature (SBS88) (81). This strain synthesizes the genotoxin colibactin, which induces host cell DNA damage through characteristic DNA mutations including single-base substitutions and insertion-deletions (81, 82). In human intestinal organoid models, pks⁺ *E. coli*-mediated DNA mutations are predominantly T>N single-base substitutions, with particular enrichment at ATN and TTT trinucleotide sequences (81). Clinical studies have corroborated these DNA damage events, demonstrating that pks⁺ *E. coli* can drive somatic mutations in the adenomatous polyposis coli (APC) gene (c.835–8 A>G), an early genetic event in CRC development (82). Further analyses have revealed that pks⁺ *E. coli* is significantly enriched in CRC tumor tissues and closely associated with elevated rates of genomic insertion-deletions, which are intimately linked to CRC-associated DNA driver mutations (83). Moreover, microbial synergistic interactions can exacerbate DNA damage. In murine models, dual infection with pks⁺ *E. coli* and ETBF results in more severe colonic epithelial DNA damage, accelerated tumorigenesis, and significantly increased mortality compared with single-pathogen infection. Mechanistically, ETBF facilitates pks⁺ *E. coli* intestinal colonization, synergistically enhancing colonic epithelial DNA damage (39).

Colibactin is also a critical mediator of DNA damage. pks⁺ *E. coli* synthesizes this genotoxin, which mediates DNA alkylation at adenine residues (84), resulting in DNA double-strand breaks (DSBs) and cell cycle arrest (85), ultimately leading to uncontrolled cell proliferation and malignant transformation (81). Arthur et al. demonstrated that deletion of the pks genotoxicity island in *E. coli* significantly reduced tumor incidence and invasiveness in mice without appreciably affecting intestinal inflammation (86). This study indicates that the toxic effects of the pks genotoxicity island drive CRC development through direct DNA damage. Microbial genomic analyses further demonstrated that the carcinogenic activity of pks⁺ E. coli is critically inflammation-dependent, with distinct genomic signatures distinguishing carcinogenic from non-carcinogenic strains in the inflamed colonic microenvironment (86). *E. coli* induces host DNA DSBs through copper-mediated oxidative cleavage reactions and exacerbates DNA damage via the IL-17 pathway, driving tumorigenesis (39). Colibactin forms complexes with intraluminal copper ions and, in concert with molecular oxygen in epithelial cells, generates ROS that further attack and cleave DNA (87). This process triggers DNA damage and promotes CRC pathogenesis. Furthermore, BFT produced by ETBF plays an important role in CRC pathogenesis. Boleij et al. reported that BFT exposure is a significant risk factor for advanced CRC, with detection rates substantially higher in late-stage than in early-stage patients (88). BFT upregulates SMOX expression, generating ROS that further promote DNA damage and drive tumor formation and progression (78). Different microorganisms play important roles in the cumulative DNA damage of host cells through multiple pathways, including genotoxin production and interference with DNA repair mechanisms, providing the molecular basis for CRC initiation, progression, and metastasis.

### 3.5 Intratumoral microbiota and non-coding RNA

Intratumoral microbiota exert critical regulatory effects on CRC progression through their interactions with ncRNAs. These microorganisms influence not only tumor cell proliferation and apoptosis but also key biological processes including migration and metastasis. Intratumoral microbiota modulate the gene expression profiles of CRC cells by affecting intracellular ncRNA expression, thereby promoting CRC development (89). ETBF downregulates miR-149-3p expression in tumor cell-derived exosomes, promoting alternative splicing of the RNA splicing factor PHD finger protein 5A (PHF5A), which in turn activates superoxide dismutase 2 (SOD2), thereby enhancing CRC cell proliferative capacity (89). Additionally, ETBF-induced overexpression of *Bacteroides fragilis*-associated long non-coding RNA 1 (BFAL1) activates the Ras homolog enriched in brain (RHEB)/mTOR signaling pathway through competitive binding with miR-155-5p and miR-200a-3p, further promoting CRC tumor growth (90). These findings demonstrate that intratumoral microbiota promote tumor cell proliferation and drug resistance by altering the ncRNA regulatory network within CRC cells. Following DNA damage induction, pks⁺ *E. coli* upregulates oncogene c-MYC expression, subsequently elevating miR-20a-5p levels. miR-20a-5p binds to the 3′-untranslated region (3′-UTR) of sentrin/SUMO-specific protease 1 (SENP1), inducing translational silencing and promoting CRC progression (91, 92). Conversely, butyrate-producing bacteria inhibit c-MYC protein expression, reduce oncogenic miR-92a levels, and activate p57 translation, thereby suppressing CRC cell proliferation (93). These findings are primarily based on cell line and murine model studies; the clinical relevance of microbiota–ncRNA regulatory networks in human CRC awaits *in situ* validation through spatial transcriptomics and single-cell multi-omics approaches.

*Parvimonas micra* promotes CRC cell proliferation by activating the miR-218-5p/protein tyrosine phosphatase receptor type R (PTPRR)/MAPK signaling pathway (94). This finding further emphasizes that microorganisms directly influence tumor cell signal transduction and proliferative capacity by regulating specific ncRNAs, particularly miRNAs. *F. nucleatum*-derived Fusobacterium adhesin A (FadA) binds to E-cadherin on intestinal epithelial cells, activating β-catenin and promoting cell proliferation (95). *F. nucleatum* also suppresses CRC cell apoptosis by targeting the TLR4/MYD88/miR-18a*/unc-51-like autophagy activating kinase 1 (ULK1), TLR4/MYD88/miR-4802/autophagy-related 7 (ATG7), and miR-31 autophagy networks (96, 97). These studies reveal that *F. nucleatum* suppresses cell apoptosis and promotes tumor growth and metastasis through interactions with ncRNAs across multiple signaling pathways. EMT constitutes the pathological foundation through which epithelial-derived malignant cells acquire migratory and invasive capabilities, playing a critical role in CRC metastasis. Studies have demonstrated that intratumoral microbiota mediate EMT through regulation of ncRNA release, thereby promoting CRC metastasis. *F. nucleatum* promotes exosomal secretion of miR-122-5p, activating the fucosyltransferase 8 (FUT8)/TGF-β1/Smads axis to induce EMT and accelerate CRC metastasis (98). Furthermore, *F. nucleatum* infection stimulates tumor cells to produce exosomes enriched with miR-1246, miR-92b-3p, miR-27a-3p, and CXCL16/Ras homolog family member A (RhoA)/IL-8. These exosomes transfer from infected to uninfected cells, enhancing migratory capacity and promoting hepatic metastasis (99). These studies demonstrate that intratumoral microbiota enhance tumor cell migration and metastatic capacity through modulation of exosomal ncRNAs.

## 4 Translational applications of intratumoral microbiota in cancer diagnosis and treatment

Beyond their role as critical contributors to tumorigenesis and progression, the translational potential of intratumoral microbiota in cancer diagnosis and treatment has gained increasing recognition. As research into tumor-associated microbial communities deepens, evidence suggests that the relationship between intratumoral microbiota and tumors extends beyond disease progression to potentially offer novel avenues for early diagnosis, therapeutic intervention, and prognostic assessment (100). **Figure 3** illustrates the translational applications of intratumoral microbiota in CRC diagnosis, therapy, and prognosis.

**Figure 3:**
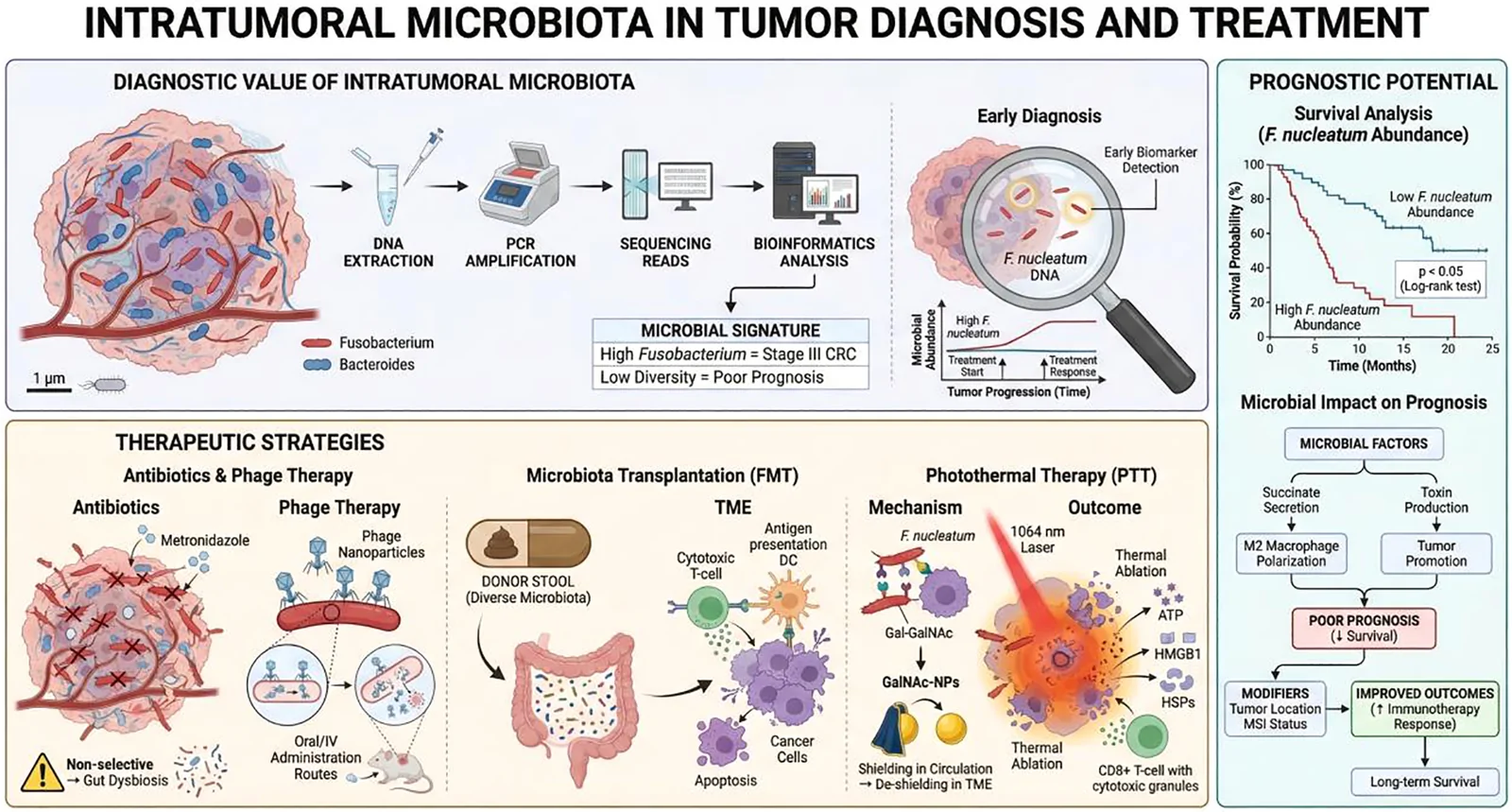
Clinical applications of intratumoral microbiota in CRC diagnosis, therapy, and prognosis. Summary of translational strategies leveraging intratumoral microbiota. Diagnostic applications: high-throughput sequencing enables construction of tumor microbial fingerprints as early diagnostic biomarkers; dynamic monitoring of microbial shifts reflects malignancy severity and therapy response. Therapeutic strategies: targeted elimination of oncogenic bacteria using antibiotics (e.g., metronidazole), phage therapy for selective F. nucleatum depletion, FMT for immune modulation, and photothermal nanotherapy exploiting F. nucleatum–Gal-GalNAc binding specificity. Prognostic value: F. nucleatum DNA abundance inversely correlates with patient survival; microbiota-based signatures predict treatment response and recurrence risk.

### 4.1 Diagnostic value of intratumoral microbiota

In recent years, intratumoral microbiota have attracted considerable attention as emerging diagnostic biomarkers for cancer. Studies have demonstrated significant differences in microbial abundance and compositional profiles across different tumor types, with these differences reflecting not only tumor type but also disease stage and prognosis (14, 101, 102). The characteristics of intratumoral microbiota can serve as potential biomarkers for early diagnosis, tumor type identification, and disease staging assessment. By analyzing microbial communities in tumor tissues using high-throughput sequencing technologies, tumor-specific microbial fingerprints can be constructed, opening new possibilities for precision cancer diagnostics (65). The most mature approach to date involves fecal multi-bacterial panel detection. A cross-continental meta-analysis by Wirbel et al. identified a 29-species core microbial set — including *F. nucleatum*, *P. micra*, *Solobacterium moorei*, and others — with best-performing models using 16–20 biomarkers achieving a diagnostic area under the curve (AUC) ≈ 0.80, comparable to FIT. However, only a few such assays have entered the market as laboratory-developed tests (LDTs), and none have received U.S. Food and Drug Administration (FDA)/National Medical Products Administration (NMPA) regulatory approval as standalone CRC screening tools, positioning this approach at an “early-to-mid translational” stage (103).

### 4.2 Therapeutic strategies targeting intratumoral microbiota

Based on existing mechanistic evidence, targeted elimination of specific intratumoral oncomicrobes (such as *F. nucleatum*, pks⁺ *E. coli*, and ETBF) may theoretically serve as an adjunctive antitumor therapeutic strategy. The vast majority of “microbiota-targeted therapies” remain at the preclinical or early clinical exploration stage, with none yet incorporated into any major guideline as standard CRC treatment. Intratumoral microbiota promote tumor initiation, progression, and metastasis by altering the TME and host immune responses. Therefore, targeted elimination or modulation of intratumoral microbial communities represents a potential adjunctive strategy for cancer therapy.

#### 4.2.1 Antibiotics and bacteriophage therapy

Antibiotics, as conventional antimicrobial therapeutic agents, also have a certain role in cancer treatment. Metronidazole has been shown to significantly reduce *Fusobacterium* burden in murine CRC models, thereby inhibiting tumor cell proliferation and CRC growth (19). However, systemic antibiotic administration can cause extensive gut microbiota dysbiosis, antibiotic-associated diarrhea, *Clostridioides difficile* infection, and dissemination of antibiotic resistance, and multiple studies have suggested that perioperative antibiotic exposure may paradoxically impair immunotherapy benefit by disrupting gut immune–microbiota homeostasis. Similarly, non-steroidal anti-inflammatory drugs (NSAIDs) such as aspirin exhibit direct antibacterial activity against certain bacteria including *F. nucleatum*, potentially reducing their enrichment in tumors and diminishing their tumor-promoting effects (104). However, the broad-spectrum antibacterial activity of antibiotics lacks selectivity and may cause gut microbiota dysbiosis, thereby affecting host immune responses. Consequently, targeted approaches directed at specific microorganisms represent a more promising strategy. Bacteriophage therapy offers high specificity, enabling precise targeting of individual bacterial species. Studies have shown that phages targeting *F. nucleatum* can effectively penetrate tumor tissues and infect target bacteria (105). Zheng et al. demonstrated that intravenous or oral administration of phage-modified nanoparticles in CRC mice significantly enhanced chemotherapeutic efficacy and reduced intratumoral *F. nucleatum* burden (106). The high specificity of phages and their nanoparticle carriers renders them promising preclinical candidates; however, challenges including *in vivo* safety (particularly endotoxin release), immunogenicity, target bacterial resistance evolution, and route/dose optimization remain to be addressed.

#### 4.2.2 FMT and microbiota modulation

Fecal microbiota transplantation (FMT) is an emerging biotherapeutic modality that restores microbial homeostasis by engrafting healthy donor microbiota into the patient’s gastrointestinal tract. Compared with conventional modulation strategies, FMT enhances microbial diversity while avoiding antibiotic-like microecological disruption. It achieves long-term microbial engraftment, overcoming the transient colonization limitations of probiotics and prebiotics (107, 108). FMT applications in CRC treatment are in the exploratory stage. Experimental studies have demonstrated that fecal transplantation from wild-type mice enhances resistance to dextran sulfate sodium/azoxymethane (DSS/AOM)-induced colorectal tumorigenesis in laboratory mice (109). This finding provides an experimental basis for FMT applications in CRC prevention and treatment; however, its molecular mechanisms and clinical translational value require further elucidation. In the CRC setting, the antitumor effects of FMT are primarily supported by murine colonization experiments and limited preclinical validation (110); no randomized controlled phase III trials have confirmed its efficacy, and substantial variability across studies in “responder donor” versus “non-responder donor” selection criteria, preparation methods, administration routes, and immune assessment timepoints precludes definitive efficacy conclusions (111). Moreover, safety concerns associated with FMT in clinical applications cannot be overlooked — in 2019, the U.S. FDA reported cases of fatal bacteremia caused by extended-spectrum β-lactamase (ESBL)-producing *E. coli* transmitted through FMT, further narrowing the safety margins for FMT in cancer patients (particularly those with neutropenia or immunosuppression). Accordingly, characterizing FMT as a “powerful tool for CRC treatment” is premature; a more accurate description would be “a promising preclinical/early-clinical candidate strategy still requiring large-scale randomized controlled trial validation”.

#### 4.2.3 Photothermal therapy

Photothermal therapy (PTT) has emerged as a novel antitumor treatment modality that has garnered considerable interest in recent years (112). PTT not only effectively suppresses primary tumors but also enhances immune responses against distant tumors by inducing immunogenic cell death (ICD). ICD releases immunogenic tumor-associated antigens and damage-associated molecular patterns (DAMPs), including adenosine triphosphate (ATP), calreticulin (CRT), high mobility group box 1 (HMGB1), and heat shock proteins (HSPs), promoting DC maturation and further enhancing CD8⁺ T cell tumor infiltration, thereby achieving tumor immune surveillance (113). Within the TME, bacterial enrichment is significantly higher than in normal tissues (114). Orally derived *F. nucleatum* exhibits selective colonization characteristics in CRC, significantly altering microbial community structures between tumor and normal tissues through interactions with CRC cell surface glycan molecules (115, 116). This property renders *F. nucleatum* a potential target for PTT. Studies have shown that *F. nucleatum* influences TIME characteristics, particularly by suppressing CD8⁺ T cell migration through its metabolite succinate, thereby attenuating antitumor immune responses (77). Additionally, the presence of *F. nucleatum* promotes M2-polarized macrophage infiltration, facilitating tumor cell immune evasion (117). These findings suggest that combining PTT with strategies targeting F. nucleatum-mediated immunosuppression may represent a promising direction for enhancing antitumor efficacy. F. nucleatum-targeted photothermal nanosystems remain a conceptual/preclinical direction, and the mechanistic claims are now explicitly framed as hypothetical pending direct validation. However, all photothermal immune antibacterial combined nanosystems described above are currently supported exclusively by preclinical data from murine models, and considerable translational distance remains before human applications; *in vivo* light penetration depth, systemic biodistribution, immunogenicity, overall effects on gut microbiota, and long-term safety all require further evaluation.

### 4.3 Prognostic potential of intratumoral bacteria

Intratumoral microbiota demonstrate significant correlations with cancer patient survival and prognosis, establishing them as potential prognostic prediction tools (11). Beyond CRC, intratumoral microbiome profiles have also been linked to immune infiltration patterns and prognosis in other gastrointestinal malignancies, such as esophageal squamous cell carcinoma (118). Particularly in CRC, the mucosal microbiome of tumors exhibits dynamic associations with disease phenotype, providing a theoretical basis for microbiome-based prognostic assessment approaches (119). Studies have found that *F. nucleatum* DNA content increases significantly with tumor progression, and this change is closely associated with decreased patient survival (66). Furthermore, *F. nucleatum* is generally considered an indicator of poor prognosis in CRC, esophageal cancer, and pancreatic cancer. The prognostic impact of *F. nucleatum* on CRC may be modulated by multiple factors (32). Their study indicated that the prognostic influence of *F. nucleatum* on CRC patients depends primarily on specific tumor location and MSI status. Tumor location and MSI status may play important roles in the mechanisms regulating the prognostic effects of *F. nucleatum* on CRC patients (31). Therefore, designating *F. nucleatum* as a universal prognostic biomarker may be overly simplistic; a more rational approach would involve microbiota–host stratified prognostic models incorporating MSI status, anatomical location, and disease stage, which represents an important direction for future cohort studies. In summary, intratumoral microbiome research has not only revealed their critical roles in tumorigenesis, progression, and immune evasion but also provided new perspectives for early cancer diagnosis, personalized treatment, and prognostic assessment. With deeper investigation into the interactions between the microbiome and the TIME, it is anticipated that more precise and effective therapeutic strategies can be developed to improve cancer patient prognosis and treatment outcomes.

## 5 Current challenges

As the intratumoral microbiome represents a quintessential low-biomass specimen, the reliability of research conclusions is critically dependent on the rigor of experimental design and data analysis. The following methodological issues directly affect the comparability of literature and credibility of conclusions in this field and must be carefully considered when interpreting study results: (1) Reagent and environmental contamination: Microbial DNA from commercial DNA extraction kits, PCR reagents, laboratory surfaces, and operator skin can generate background noise far exceeding authentic signals in low-biomass samples (120). Therefore, all intratumoral microbiome studies should include reagent negative controls (no-template controls), environmental controls, and parallel-processed adjacent normal tissue controls, with bioinformatic decontamination (e.g., decontam, SCRuB) applied during analysis. (2) Sequencing depth, database selection, and taxonomic confidence: The taxonomic resolution of 16S rRNA amplicon sequencing is limited, and metagenomic sequencing is prone to false-positive classifications at low-abundance species levels (121). Differences in database versions (SILVA, Genome Taxonomy Database [GTDB], National Center for Biotechnology Information [NCBI] RefSeq) and host-depletion strategies across studies constitute a major source of inter-cohort inconsistencies in microbial profiles for the same cancer type. Furthermore, the lack of a universally accepted definition of the ‘core’ human microbiome compounds these inconsistencies; recent proposals emphasizing relational stability across hosts may offer a more reliable framework for cross-cohort comparison of intratumoral microbial signatures (122). (3) Association versus causation: The vast majority of conclusions regarding bacterial enrichment in CRC tissues derive from cross-sectional paired tissue comparisons, representing associative evidence that cannot directly establish whether a given bacterium is a “cause” of carcinogenesis or a “consequence” of TME alterations. Establishing causal relationships typically requires germ-free or antibiotic-treated murine strain colonization–tumorigenesis experiments, Mendelian randomization analyses, or temporal exposure–outcome relationships in prospective cohorts. Among CRC-associated bacteria, F. nucleatum, pks⁺ E. coli, and ETBF currently have relatively stronger mechanistic support from organoid, animal, and human association studies. Nevertheless, whether these microorganisms act as true causal drivers, passengers, or context-dependent contributors remains to be established through longitudinal cohorts and interventional studies, consistent with the association-versus-causation framework proposed by the International Cancer Microbiome Consortium (123). (4) Batch effects and population heterogeneity: Diet, geography, antibiotic exposure history, and sample preservation duration can all significantly influence intratumoral microbial profiles, and cross-cohort meta-analyses must apply rigorous batch correction. Tito et al. demonstrated through quantitative microbial profiling that ignoring confounding factors significantly inflates the effect sizes of multiple predictive “CRC signature bacteria” (124). In particular, the network topology of gut microbiota in Chinese populations differs substantially from that in Western cohorts, and guild-based analyses indicate that predictive models constructed solely from Western databases require cautious assessment of their transferability to Chinese populations (125).

## 6 Conclusions

Intratumoral microbiota play a crucial role in CRC initiation, progression, and metastasis. A growing body of evidence indicates that microbial communities within the TME are closely linked to the host immune system, tumor immune evasion mechanisms, and processes of tumor proliferation and metastasis. Different microbial species exert specific roles across distinct stages of CRC development. For instance, pathogenic bacteria such as *F. nucleatum* and *B. fragilis* serve as important promoters of CRC by remodeling the TME, suppressing antitumor immunity, and facilitating tumor cell proliferation and metastasis. Furthermore, intratumoral microbiome compositional changes are closely correlated with different developmental stages, malignancy grades, and patient prognosis in CRC. Consequently, intratumoral microbiota not only provide new avenues for early CRC diagnosis and treatment but also represent novel therapeutic targets. Studies have also demonstrated that microbiota-targeted therapeutic strategies — including antibiotics, bacteriophage therapy, and FMT — have yielded preliminary translational results, effectively suppressing tumor growth and enhancing immunotherapeutic efficacy. Bacteriophage therapy, with its high species-level specificity, has shown unique advantages in cancer treatment. Additionally, the combination of PTT with microbiome modulation offers novel strategies for enhancing tumor immunogenicity and improving therapeutic outcomes.

However, alongside these advances, the fundamental limitations persisting in this field must be clearly acknowledged: (1) most associative evidence derives from cross-sectional paired tissue sequencing, with incomplete causal chains — whether specific oncomicrobes are “drivers” or “passengers” may vary across different bacteria and disease stages; (2) the low-biomass nature of intratumoral microbiome specimens renders contamination and batch effects the primary sources of reproducibility crises, urgently requiring standardized sample collection, negative control, and bioinformatic decontamination protocols; (3) mechanistic studies predominantly rely on murine models, yet significant compositional differences between murine and human gut microbiota necessitate cautious cross-species extrapolation; (4) the vast majority of proposed therapeutic strategies (including targeted antibiotics, bacteriophage therapy, FMT, and photothermal antibacterial nanosystems) remain at the preclinical or early clinical stage, with none incorporated into major clinical guidelines as standard CRC treatment; and (5) although microbiome-based diagnostic panels have demonstrated performance comparable to FIT in retrospective cohorts, their transferability across geographically and dietarily diverse populations remains to be validated. Breakthroughs in this field will likely depend on integrated advances across several key directions: first, establishing and promoting international standardized protocols for intratumoral microbiome research — rigorous implementation of guidelines such as Strengthening The Organization and Reporting of Microbiome Studies (STORMS) will substantially improve literature comparability; second, developing spatial transcriptomics, spatial microbiomics, and single-cell multi-omics integrated analyses to precisely characterize microbe–host cell interactions *in situ*; third, conducting causal inference studies based on Mendelian randomization, prospective cohort designs, and randomized controlled intervention trials to convert associative evidence into causal evidence; fourth, identifying precision therapeutic breakthroughs at the metabolism–microbiome–immunity intersection, with glutamine metabolism, bile acid metabolism, and the tryptophan–AhR axis representing compelling integrative targets; and fifth, at the translational level, designing clinical trials that employ microbiome-based biomarker enrichment strategies rather than uniformly applying FMT or antibiotics to all CRC patients, thereby truly realizing “microbe–host–tumor” three-dimensional precision oncology. In conclusion, the relationship between intratumoral microbiota and CRC provides not only novel biomarkers but also fresh perspectives for personalized cancer therapy. However, translating these mechanistic discoveries into safe, effective, and scalable clinical tools remains a long-term endeavor demanding rigorous methodology, systematic evidence generation, and multidisciplinary collaboration.
